# Treatment of Large Incisional Hernias in Sandwich Technique - A Review of the Literature

**DOI:** 10.3389/fsurg.2018.00037

**Published:** 2018-05-28

**Authors:** Ferdinand Köckerling, Hubert Scheuerlein, Christine Schug-Pass

**Affiliations:** ^1^Department of Surgery and Center for Minimally Invasive Surgery, Academic Teaching Hospital of Charité Medical School, Vivantes Hospital, Berlin, Germany; ^2^Department of General and Visceral Surgery, St. Vinzenz Hospital, Paderborn, Germany

**Keywords:** sandwich technique, double mesh technique, wound complications, recurrence, incisional hernia

## Abstract

**Introduction:**

In a systematic review of the surgical treatment of large incisional hernia sublay repair, the sandwich technique and aponeuroplasty with intraperitoneal mesh displayed the best results. In this systematic review only the sandwich technique, which used the hernia sac as an extension of the posterior and anterior rectus sheath and placement of a non-absorbable mesh in the sublay position, was included. Other modifications of the sandwich technique are published in the literature and were also analyzed in this literature review.

**Methods:**

A systematic search of the available literature was performed in November 2017 using Medline, PubMed, and the Cochrane Library using the terms “sandwich technique”, “double prosthetic repair”, “double mesh intraperitoneal repair”, and “component separation technique with double mesh”. This review is based on 24 relevant publications. Unfortunately, the evidence of the available studies is not very high since only prospective and retrospective case series have been published. There are no comparative studies at all. Therefore, the findings of the published case series must be viewed in a critical light.

**Results:**

The published studies report a remarkably low recurrence rate of 0-13% with a follow-up of 1–7 years. One limitation that must be mentioned here is that in around half of the studies the method of follow-up was not specified and in the remaining cases this was based on clinical examination by the surgical team. This puts into perspective the reported results, which appear to be too favorable given the complex nature of the hernias involved.

The major disadvantage of the sandwich technique is a very high rate of wound complications of up to 68%, mainly induced by creation of large skin and subcutaneous cellular tissue flaps.

**Conclusion:**

It is difficult to evaluate the significance of the various modifications of the “sandwich technique” based on the available literature since it includes only case series and no comparative studies. The techniques used are associated with very high wound complication rates but with only relatively low recurrence rates despite the complexity of the cases involved. This must be verified in studies with a well-designed methodology.

## Introduction

According to the European Hernia Society classification of incisional abdominal wall hernias, the largest defects have a width of 10 cm or more (1). A systematic review of the surgical treatment of large incisional hernias included studies describing patients with an incisional hernia with a diameter of 10 cm or a surface of 100 cm^2^ or more (2). “Recurrence hazards per year were calculated for all techniques using a generalized linear model” (2). Fifty-five articles were included, containing 3,945 large incisional hernia repairs (2). “Mesh reinforced techniques displayed better recurrence rates and hazards than techniques without mesh reinforcement, including component separation technique without mesh” (2). Of all the mesh techniques, including component separation technique with mesh, sublay repair, the “peritoneal sandwich technique” with sublay mesh and aponeuroplasty with intraperitoneal mesh displayed the best results with recurrence rates of <3.6% and recurrence hazard <0.5% per year (2).

In the systematic review only the sandwich technique, which used the hernia sac as an extension of the posterior rectus sheath and the anterior rectus sheath (“peritoneal sandwich technique”) and placement of a non-absorbable mesh in the sublay positon, was analyzed (2).

However, in the literature under the terms “sandwich technique”, “double prosthetic repair”, “component separation technique with double mesh” and “double mesh intraperitoneal repair”, there are several technical variations that may be summarized under the heading “sandwich technique”. A common feature of these techniques is that they differ from the standard techniques (laparoscopic intraperitoneal onlay mesh, sublay, component separation technique, open intraperitoneal onlay mesh, onlay, inlay) and can be used for large incisional hernias.

Based on the literature review, the various types of “sandwich technique” and associated outcomes were analyzed and are now presented below.

## Methods

In the literature search the following terms were used: “hernia and sandwich technique” (*n* = 13), “hernia and double prosthetic” (*n* = 44), “component separation technique with double mesh” (*n* = 2), “double mesh intraperitoneal repair” (*n* = 15). The systematic search of the available literature was performed in November 2017 using Medline, PubMed, and the Cochrane Library, as well as a search of relevant journals and reference lists using the aforementioned search terms. This review is based on 24 relevant publications. Of these, 15 publications with study results report on three prospective and 12 retrospective case series. No comparative studies were included. Furthermore, very different surgical techniques were classified under the collective term “sandwich technique” or “double mesh technique”. That further limits comparability with the standard incisional hernia surgical techniques. Therefore, the published case series reported on below can only be classified into groups on the basis of the techniques used and the results then presented separately.

## Results

### Peritoneal Sandwich Technique

In the systematic review by Deerenberg et al. (2) only the results of the “sandwich technique” which used the hernia sac as an extension of the posterior and anterior rectus sheath were analyzed. A non-absorbable mesh is implanted in the sublay position to reinforce the repair. As such, this constitutes a modification of the sublay technique.

**Figure 1:**
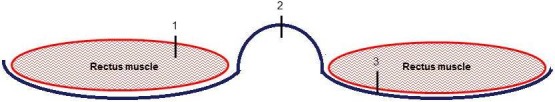
Peritoneal sandwich technique. Incision of the anterior rectus sheath (1), the hernia sac (2) and the posterior rectus sheath (3) Red = Rectus sheath; Blue = Peritoneum and hernia sac.

**Figure 2:**
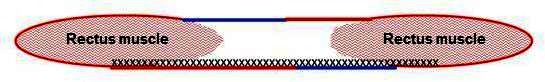
Mesh placement posterior to the rectus muscles on the reconstructed posterior layer of the rectus sheath. Red = Rectus sheath; Blue = Peritoneum and hernia sac.

Part of the anterior layer of the left or right rectus sheath and of the corresponding hernia sac is used for reconstruction of the posterior layer of the rectus sheath (Figure 1). The mesh is positioned posterior to the rectus muscle and placed on the reconstructed posterior layer of the rectus sheath (Figure 2). To cover the mesh the anterior layer of the rectus sheath is reconstructed with part of the posterior layer of the rectus sheath and the corresponding hernia sac.

Hence, this gives rise to a partial bridging situation, i.e., there remains a partial defect that is only closed with the mesh and an anterior and posterior portion of the hernia sac. This means that the mesh positioned behind the rectus muscle is only partially covered at the front and back by the peritoneum of the hernia sac (“peritoneal sandwich technique”). In studies, large incisional hernias were repaired using this technique (2).

Matapurkur et al. (3) published a case series of 60 patients with large incisional hernias treated over 15 years. Marlex polyprophylene mesh was used for repair, sandwiched between two layers of peritoneum of the overstretched hernia sac. There was no operative mortality and no recurrence in follow-up from three years to seven years. Postoperative complications were major wound infection in four patients (6.7%), minor wound infection in six patients (10%), one patient had local discomfort and pain and two suffered from delayed peristalsis. “Postoperative complications encountered could be managed conservatively, without necessitating any major surgical procedure or mesh removal” (3). No recurrence was noted in these patients (3, Table 1).

**Table 1:** Study results with peritoneal sandwich technique.

Author	Study design	n	Follow-up	Method of follow-up	Complications	Recurrence rate	Type of mesh 1	Type of mesh 2	Conclusion
Matapurkar (3)	Retrospective case series	60	3–7 y	Not specified	Minor wound infection n = 6, major wound infection n = 4, local discomfort and pain n = 1, delayed peristalsis n = 2	0 %	Polypropylene (Marlex)	–	Postoperative complications encountered could be managed conservatively without mesh removal
Martinez (4)	Retrospective case series	53, only 11 cases in peritoneal sandwich technique	Mean follow-up 40 months (range 6–78 months)	Not specified	Wound infection n = 2 (3.7%)	7.5 %	ePTFE	–	–
Katsaragakis (5)	Retrospective case series	19	Mean follow-up 23 months (range 9–45 months)	Clinical examination by the surgical team and an independent physician; CT, when clinical examination was doubtful	Wound infection n=2	0 %	Polyester (Mersilene)	–	Advantages of this technique: viscera are protected from direct mesh contact, mesh is protected during wound infection, reduction of intraabdominal pressure
Bracci (6)	Retrospective case series	26	24 months	Review at distance by the surgical team	Seroma n=2	0 %	Polypropylene	–	Useful technique
Malik (7)	Retrospective case series	21	Mean follow-up 37 months	Clinical examination by the surgical team (n = 14) and by telephone (n = 5). Missing patients (n = 2)	Superficial skin necrosis n = 3, superficial wound infection n = 2, seroma n = 2, abdominal wall necrosis n = 1	n = 1 (4.8%)	Polypropylene	–	Useful method for repairing large ventral hernias

Martinez et al. (4) used in 53 cases with large incisional hernias expanded polytetrafluoroethylene meshes as replacement of the abdominal wall, in 11 cases in peritoneal sandwich technique. They reported two recurrences in the series of 53 operations (7.5%) and wound infection in two cases (3.7%).

In a case series of 19 patients with large incisional hernias, Katsaragakis et al. (5) used the peritoneal sandwich technique with a polyester mesh (Mersilene). Two patients (10.5%) developed wound infections that were easily treated conservatively. They had no seromas or other complications. The mean follow-up time was 23 months (range 9–45 months) and no recurrence or late complication has been mentioned.

Bracci et al. (6) studied 26 patients operated on with the “peritoneal sandwich technique” using a polypropylene mesh. The follow-up was 24 months. No prosthetic infections were reported and no recurrence was observed. They had two cases of seroma, treated conservatively.

In a case series published by Malik et al. (7) twenty-one ventral and incisional hernias were treated with the “peritoneal sandwich technique” and polypropylene mesh in sublay position. Defect size ranged from 25 to 500 cm^2^ and mesh sizes from 300 to 900 cm^2^. Eighteen were incisional hernias (13 midline, three transverse and two oblique incisions), and three were primary paraumbilical hernias. Three cases of superficial skin edge necrosis, two superficial wound infections and two sizeable seromas developed (surgical site occurrence *n* = 7; 33.3%), but all had resolved within six months. One patient developed abdominal wall necrosis requiring mesh removal and reconstruction without mesh, resulting in late recurrence. All others achieved excellent long-term outcomes.

### Double Mesh Sandwich Techniques

Unlike in the “peritoneal sandwich technique”, where a mesh is positioned between two peritoneal layers, in the “double mesh sandwich technique” two meshes are used for defect closure of large incisional hernias. In that respect, a distinction is made between the “double intraperitoneal onlay mesh technique”, “double onlay mesh technique”, “double underlay and onlay mesh technique” and the “component separation with double mesh technique”.

#### A. Double intraperitoneal onlay mesh technique

**Figure 3:**
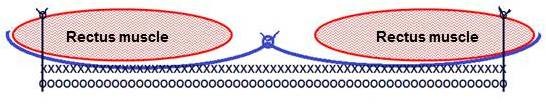
Double intraperitoneal onlay mesh technique Red = Rectus sheath; Blue = Peritoneum.

In the intraperitoneal double mesh sandwich technique two meshes are placed in the open intraperitoneal onlay technique for closure of large incisional hernias (Figure 3). Afifi (8) reported on 19 patients with a large recurrent ventral hernia operated on with this technique. The cutaneous scar was excised and the hernia sac dissected. “The sac was opened and any intraperitoneal adhesions, especially those related to the inner aspect of the anterior abdominal wall, were divided. The rectus sheath and the external oblique aponeurosis were exposed for a minimum distance of 10 cm around the defect from all directions” (8). The mesh was prepared by suturing a Vicryl and polypropylene mesh together. The mesh was inserted intraperitoneally with the Vicryl part facing the bowel. U-sutures were applied through the whole thickness of the anterior abdominal wall in order to anchor the mesh. The sac was closed over the mesh.

Postoperative complications reported were superficial wound infection in one case (5.8%), deep vein thrombosis and fatal pulmonary embolism in one case (5.8%), and pain in six patients (35.3%). In a mean follow-up of 30 months no recurrence was identified.

#### B. Double onlay mesh technique

In a review article Moreno-Egea et al. (9) give an historic overview explaining the development of the double mesh sandwich techniques. The authors additionally describe the intra- and extraperitoneal double mesh sandwich technique in their publication. This technique was inaugurated by Usher et al. (10).

**Figure 4:**
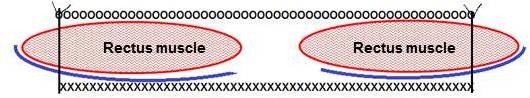
Double onlay mesh technique Red = Rectus sheath; Blue = Peritoneum.

“The scar is resected and two wide flaps of skin and subcutaneous cellular tissue are dissected, widely surpassing the hernia defect using an electric scalpel and exposing the fat-free aponeurosis of the external oblique muscle in order to facilitate later contact with the mesh” (9). The peritoneal cavity is opened and complete adhesiolysis is performed. For the first repair, a large mesh recommended for intraabdominal use is placed behind the parietal wall and circumferentially fixed with U-sutures (Figure 4). The second repair involves the use of another large mesh with supra-aponeurotic placement and fixation (9).

Moreno-Egea et al. (11) reported on a prospective case series of 50 patients with complex incisional hernias with defect sizes > 15 cm undergoing repair in double onlay mesh sandwich technique using Proceed™ and a low weight polypropylene mesh. In the postoperative course five patients (10%) suffered from a seroma, two (4%) from skin necrosis and one (2%) from wound infection. In a mean follow-up of 48 months no recurrence was detected. (Table 2)

**Table 2:** Study results with double onlay mesh technique.

Author	Study design	n	Follow-up	Method of follow-up	Complications	Recurrence rate	Type of mesh 1	Type of mesh 2	Conclusion
Moreno-Egea (11)	Prospective case series	50	Mean follow-up 48 months (range 12-108 months)	Clinical examination by the surgical team, CT when suspicion of a recurrence	Seroma n=5, neuralgia n=2, cutaneous necrosis n=2	0 %	Proceed^TM^	Polypro-pylene	Complex incisional hernias can be repaired safely and with a low morbidity and recurrence rate
Rubio (12)	Retrospective case series	18	–	Not specified	Seroma n=1 with mesh removal, superficial wound infection n=1	–	PTFE	PTFE	This alternative technique provides definitive repair in difficult cases

Rubio (12) presented the results of 18 patients with giant ventral hernias operated on with double onlay mesh sandwich technique using two expanded polytetrafluoroethylene (PTFE) meshes. One patient developed a seroma which led to removal of the extraperitoneal PTFE mesh. The patient was placed on long-term antibiotic coverage and subsequently did well.

#### C. Double Underlay and Onlay Mesh Technique

**Figure 5:**
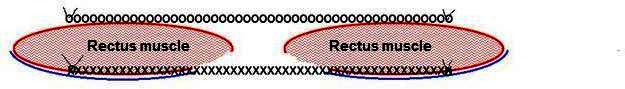
Double underlay and onlay mesh technique Red = Rectus sheath; Blue = Peritoneum.

Two other publications report on a case series using two meshes, i.e., a biologic and a synthetic mesh. Here the biologic mesh is placed in an underlay position and is in contact with the abdominal intestines in the region of the bridging (Figure 5). The authors refer to a combination of underlay with onlay technique. The biologic mesh is fixed to the posterior aspect of the anterior abdominal wall using an underlay technique. The synthetic mesh is placed in an onlay position on the anterior layer of the rectus sheath and fixed to the anterior oblique muscle.

Shaikh et al. (13) presented the results of 10 cases repaired with a Permacol™ biologic mesh and a Premilene™ polypropylene mesh. Two patients developed wound infection (20%) and one a seroma (10%). No case of recurrence was noted after a median follow-up of 15.5 months. (Table 3)

**Table 3:** Study results with double underlay and onlay mesh technique.

Author	Study design	n	Follow-up	Method of follow-up	Complications	Recurrence rate	Type of mesh 1	Type of mesh 2	Conclusion
Shaikh (13)	Prospective case series	10	Median 15.5 months (range 6-29 months)	Clinical examination by the surgical team	Wound infection n=2, seroma n=1	0 %	Permacol^TM^	Premilene^TM^	The use of double layer of porcine acellular dermal collagen implant and polypropylene mesh in reconstruction of abdominal wall defects can be considered as safe and effective
Azar (14)	Retrospective case series	21,17 in sandwich technique with additional negative – pressure wound vacuum	Mean follow-up 439 ± 310 days	Not specified	Morbidity 29 %, surgical site infection 10 %, seroma 29 %, wound dehiscence 19 %, enterocutaneous fistula 9 %, reoperation 33 %	10 %	Surgimend^TM^	Prolene^TM^	The results suggest that the sandwich technique is a safe and durable technique, even in patients with giant ventral hernias

In a study by Azar et al. (14) 17 from 21 (81 %) patients with loss of domain hernias underwent a sandwich technique using Surgimend™ acellular dermal matrix and Prolene™ polypropylene mesh with additional negative-pressure wound vacuum. Ten patients had a hernia sac-to-abdominal cavity defect less than 30%, and 11 had defects greater than 30%. There were two recurrences (18%) in the giant ventral hernia group and none in the smaller defect group. Surgical site occurrences were noted in 48% of patients and did not differ between giant and non-giant ventral hernia group (50% vs 45%; *p* = 0.84). The authors concluded that the sandwich technique is a safe and durable method to restore abdominal wall integrity.

#### D. Component separation with double mesh technique

**Figure 6:**
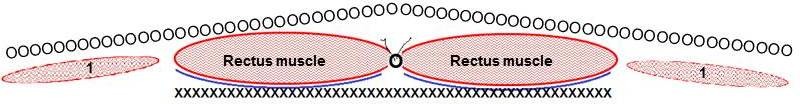
Component separation with double mesh technique Red = Rectus sheath; Blue = Peritoneum; 1 = Oblique external muscle.

Nasajpour et al. (15) presented a case series of 18 patients with a complex abdominal wall hernia treated with component separation technique paired with intra- peritoneal Collamend™ acellular porcine dermis and Prolene™ or Ultrapro™ (polypropylene) mesh overlay, covering the entire anterior abdominal wall (Figure 6).

Following anterior component separation a long-lasting biologic mesh was placed intraperitoneally. After closure of the midline a heavy-weight or light-weight polypropylene mesh was sutured over the anterior abdominal wall covering the defect of anterior oblique muscle separation. There were no hernia recurrences during the mean follow-up of 14 months. But there was a high postoperative complication rate with seroma in 33% of cases, postoperative infection requiring surgical intervention in 39% and postoperative infection requiring intervention in the preoperative infected group in 67%. The authors concluded that this technique has not led to any recurrence to date, but is prone to complications. (Table 4)

**Table 4:** Study results with component separation and double mesh technique.

Author	Study design	n	Follow-up	Method of follow-up	Complications	Recurrence rate	Type of mesh 1	Type of mesh 2	Conclusion
Nasajpour (15)	Prospective case series	18	Mean follow-up 14 months (range 4–24 months)	Clinical examination by the surgical team	Seroma 33 %, infection requiring surgical intervention 39 %	0 %	Collamend^TM^	Prolene^TM ^or Ultrapro^TM^	Mesh used in conjunction with component separation technique have a low recurrence rate, but are prone to complications
Morris (16)	Retrospective case series	51	Mean follow-up 20.6 ± 2.1 months	Clinical examination by the surgical team	Surgical site occurrences 39 % , most commonly from skin necrosis	3.9 %	Collamend^TM^	Ultrapro^TM^ orSoftMesh^TM^	Repair of large ventral hernias with CST with double mesh results in lower recurrence rates compared to historical reports of CST alone
Hicks (17)	Prospective case series	60	Median follow-up 12 months (range 5.8–26.5 months)	Not specified	Major morbidity 23.3 %, incidence of surgical site occurrence 21.7 %, surgical site infection 6.7 %	13.3 %	Surgimend^TM^	Prolene^TM^	Use of a dual layer sandwich repair with CST for complex abdominal wall repair is associated with a low recurrence rate
Martin-Cartes (18)	Retrospective case series	30	Mean follow-up 30.1 months	Not specified	Seroma 15 %, surgical site occurrence 18.9 %, reoperation rate 13.3 %	–	BioA^TM^	Optilene elastic^TM^	Complex abdominal wall defects can be successfully treated using a CST with double mesh
Bröker (19)	Retrospective case series	9	Median follow-up 13 months (range 3-49 months)	Not specified	Wound infection 44 %, total 66 %	0 %	Vypro^TM^ or Parietex composite^TM^	Vypro^TM^	The CST with double mesh has shown a low recurrence rate, but high wound infection rate
Satterwhite (20)	Prospective case series	19	Mean follow-up 11 months (range 1-33 months)	Not specified	Total n=10/19 (52.6 %), seroma n=2, wound infection n=2, abscess n=1, skin necrosis n=6, fistula n =3, reoperation n = 7/19 (36.8 %)	0 %	Permacol^TM^	Permacol^TM^	A CST with an underlay and onlay of crosslinked porcine xenograft should be considered to minimize risk of recurrence

Morris et al. (16) published a case series of 51 patients with large hernia defects undergoing a component separation technique with an intraperitoneal Collamend™ acellular porcine dermal collagen mesh and an onlay light-weight polypropylene mesh. Following placement of the biologic mesh intraperitoneally and midline abdominal closure, the polypropylene mesh (Ultrapro™, Soft Mesh™) was placed over the anterior component separation defect in the superficial oblique muscle in onlay position. Surgical site occurrences were identified in 39% of cases, most commonly from skin necrosis. Hernia recurrence rate was 3.9%. The authors concluded that repair of large, complex abdominal wall hernias by component separation technique, augmented with an intraperitoneal biologic mesh and a light-weight polypropylene onlay mesh, results in lower recurrence rates compared to historical reports of component separation technique alone.

Hicks et al. (17) performed standard anterior component separation in 60 patients. “The hernia sac was entered, and the abdominal wall was separated from the underlying viscera” (17). “The hernia sac was excised back to the edge of normal fascia” (17). A Surgimend™ biologic mesh underlay, made from acellular dermal matrix, extending 4 cm circumferentially beyond the facial edge of the defect was then sutured in place using interrupted 1 Prolene sutures. Subsequently, a soft Prolene™ polypropylene mesh onlay was used in all cases to cover the abdominal wall. A negative-pressure wound management system was then placed. At 30 days postoperatively, major postoperative morbidity occurred in 14 (23.3 %) patients, but the incidence of surgical site occurrences (*n* = 13; 21.7%) and surgical site infections (*n* = 4; 6.7%) was low. All surgical site infections were superficial. At a median follow-up of 12 months, eight patients (13.3%) had a documented hernia recurrence.

In an attempt to reduce recurrences Martin-Cartes et al. (18) frequently added a biologic (BioA) underlay mesh and a light-weight polypropylene (Optilene Elastic) onlay mesh to the traditional component separation technique. At a mean follow-up time of 30.1 months, most of the 30 patients had successful outcomes, one patient died from multi-organ failure unrelated to hernia repair.

Bröker et al. (19) performed in nine patients a component separation technique combined with a double mesh repair for large midline incisional hernia repair. After dissection of the aponeurosis of the external oblique muscle, the rectus abdominus muscle could be medialized 6–7 cm on both sides. The remaining defect in the midline was closed using a Vypro mesh. This mesh was placed preperitoneally and attached bilaterally to the rectus muscle with a 3 cm overlap of the border of the freed oblique muscle. In four patients it was not possible to close the peritoneal sac, so intraperitoneal Parietex was used instead of Vypro. On top of the Vypro or Parietex mesh, Vypro mesh was placed as an onlay to cover the previous repair (19). Postoperative complications occurred in 66%. The overall wound infection occurrence rate was 44%. Follow-up (median 13 months, range 3–49 months) showed no recurrent hernia (19).

Satterwhite et al. (20) performed in 19 patients a component separation technique with dual layer cross-linked porcine dermal xenograft. “The external oblique aponeurosis was identified on both sides, and an incision was made longitudinally to separate the external oblique muscular insertions into the rectus” (20). “An incision along the posterior rectus sheath was made 2 cm lateral to the midline, creating an edge to suture the biologic mesh (Permacol™) underlay” (20). After total or near-total fascial closure, an onlay mesh of Permacol™ was secured laterally on the external oblique muscle (20). Postoperative complications were observed in ten out of 19 patients (52.6%). Complications included seroma (*n* = 2), wound infection (*n* = 2), abscess (*n* = 1), skin necrosis (*n* = 6), and fistula formation (*n* = 3). Seven patients required re-operation (36.8%). There was no hernia recurrence in a mean follow-up of 11 months (range 1–33 months) (20).

## Discussion

The term “sandwich technique” is a collective term denoting a number of different techniques used for reconstruction of the abdominal wall in the presence of large incisional hernias. A common feature of these techniques is that they are used for large incisional hernias where the classic open repair methods, such as the sublay technique, the open IPOM technique and the component separation technique, have proved to be limited. In any case, the laparoscopic intraperitoneal onlay mesh (IPOM) technique is recommended in the guidelines only for defect sizes of up to 10 cm (21–23). The rationale for using two meshes or one mesh and the hernia sac is typically the desire to achieve better stabilization of a bridging situation or defect closure, and thus prevent a recurrence, following a component separation technique. This technique is thus reserved for very large defects. The surprisingly low recurrence rate reported to date in the studies published on this topic does indeed appear to confirm this. The recurrence rates in the published studies are between 0–13.3%, with most studies reporting a recurrence rate of 0%. Hence, the use of two meshes or one mesh and the hernia sac in a bridging situation or a component separation technique seems to reduce the recurrence rate. However, caution is advised when interpreting the results since all of the published studies were case series and there were no comparative investigations. Another limitation that must be pointed out is that in eight of 15 studies no details of how follow-up was conducted are given. Apart from one publication (5), follow-up examinations were not carried out by independent investigators. Therefore, against that background a recurrence rate of 0% must be viewed in a somewhat critical light given the complex nature of the incisional hernias involved. As such, the role of the “sandwich techniques” for repair of large incisional hernias compared with that of the standard techniques can only be evaluated to a very limited extent. Comparative studies are urgently needed. This is important in the light of the promisingly low recurrence rates for incisional hernias repaired with the “sandwich technique”.

A disadvantage of the sandwich technique is the very high wound complication rate. This is no doubt linked to the creation of wide skin flaps on dissection of the skin with the subcutaneous cellular tissue from the anterior layer of the rectus sheath and the external oblique muscle for placement of the onlay meshes. For that reason the guidelines, too, advise against the use of the “classic anterior component separation” with and without a mesh, suggesting that preference be given instead to an endoscopic and posterior component separation technique (24). This raises the question as to whether there are other potential modifications of the “sandwich technique” that will assure a comparatively low recurrence rate but also reduce the high wound complication rate. A conceivable solution here would be to place one mesh in the IPOM position with a second mesh in the retrorectal or underlay position in settings where defect closure with sublay repair or posterior component separation is not possible. Another alternative would be open IPOM repair with defect closure assured by a second mesh in the inlay position. Such studies should be conducted in the future.

## Author Contributions

FK: literature search, literature analyses, publication concept, publication draft. HS: literature search, literature analyses, publication concept, critical review of the publication draft. CS-P: literature search, literature analyses, publication concept, critical review of the publication draft.

## Conflict of Interest Statement

The authors declare that the research was conducted in the absence of any commercial or financial relationships that could be construed as a potential conflict of interest.
